# From Immunity to Neurogenesis: Toll-like Receptors as Versatile Regulators in the Nervous System

**DOI:** 10.3390/ijms25115711

**Published:** 2024-05-24

**Authors:** Daniela Melissa Abarca-Merlin, J. Abigail Martínez-Durán, J. David Medina-Pérez, Guadalupe Rodríguez-Santos, Lourdes Alvarez-Arellano

**Affiliations:** 1 Laboratorio de Investigación en Neurociencias, Hospital Infantil de México Federico Gómez, Av. Dr. Márquez 162. Colonia Doctores, Mexico City 06720, Mexico; melyabarca@gmail.com (D.M.A.-M.);; 2 CONAHCYT-Hospital Infantil de México Federico Gómez, Mexico City 06720, Mexico

**Keywords:** TLR, brain, neurogenesis, blood–brain barrier, behavior, cognition, neurodegeneration, neuroinflammation

## Abstract

Toll-like receptors (TLRs) are among the main components of the innate immune system. They can detect conserved structures in microorganisms and molecules associated with stress and cellular damage. TLRs are expressed in resident immune cells and both neurons and glial cells of the nervous system. Increasing evidence is emerging on the participation of TLRs not only in the immune response but also in processes of the nervous system, such as neurogenesis and cognition. Below, we present a review of the literature that evaluates the expression and role of TLRs in processes such as neurodevelopment, behavior, cognition, infection, neuroinflammation, and neurodegeneration.

## 1. Introduction

The activation of immune system cells is based on the recognition of exogenous molecules from organisms called microorganism-associated molecular patterns (MAMPs), as well as the recognition of endogenous molecules considered warning signals called damage-associated molecular patterns (DAMPs). The recognition of MAMPs and DAMPs is accomplished by germline-encoded receptors called pattern recognition receptors (PRRs), and this family of receptors includes Toll-like receptors (TLRs) [1]. TLRs are type I transmembrane glycoproteins that are characterized by an extracellular domain with leucine-rich repetitive regions (LRRs); MAMPs and DAMPs are recognized through this domain. They also have an intracellular domain similar to that of the interleukin I receptor (IL-1R) family called Toll/IL-1R (TIR), through which they initiate downstream signaling processes. At present, 13 members of this family have been identified in mammals, 10 of which are functional in humans (TLR1–TLR10) and 12 in mice (TLR1–TLR9 and TLR11–TLR13). TLRs can be subdivided based on their predominant cellular localization. The group of TLRs expressed on the cell membrane includes TLR1, TLR2, TLR4, TLR5, TLR6, and TLR10, while TLR3, TLR7, TLR8, and TLR9 are mainly expressed in membranes of intracellular compartments, such as endosomes, lysosomes and the endoplasmic reticulum [1].

TLR2 forms heterodimers with TLR1, TLR6, and TLR10, and in conjunction, these recognize a wide variety of MAMPs and DAMPs, such as lipopeptides, glycolipids, peptidoglycans, lipoteichoic acid, and zymosan. Among the most important described TLR3 agonists are double-stranded RNA (dsRNA) and polyinosinic-polycytidylic acid [poly(I:C), a synthetic agonist]. TLR4 recognizes lipopolysaccharide (LPS), heat shock proteins, and components of the extracellular matrix, and TLR5 recognizes flagellin. TLR7 recognizes single-stranded RNA (ssRNA), microRNAs (miRNAs), small interfering RNAs (siRNAs), and synthetic agonists, such as imidazoquinoline derivatives, including imiquimod and resiquimod (R848) and guanine analogs, such as loxoribine. TLR8 is structurally similar to TLR7 and preferentially recognizes bacterial RNA, viral dsRNA, and synthetic agonists, such as R848. TLR9 recognizes DNA with unmethylated cytosine–guanine (CpG) motifs, synthetic CpG oligonucleotides (ODNs), and immunoglobulin–DNA complexes, and TLR10 has been reported to be activated by proteins of a viral origin [1,2,3].

**Figure 1:**
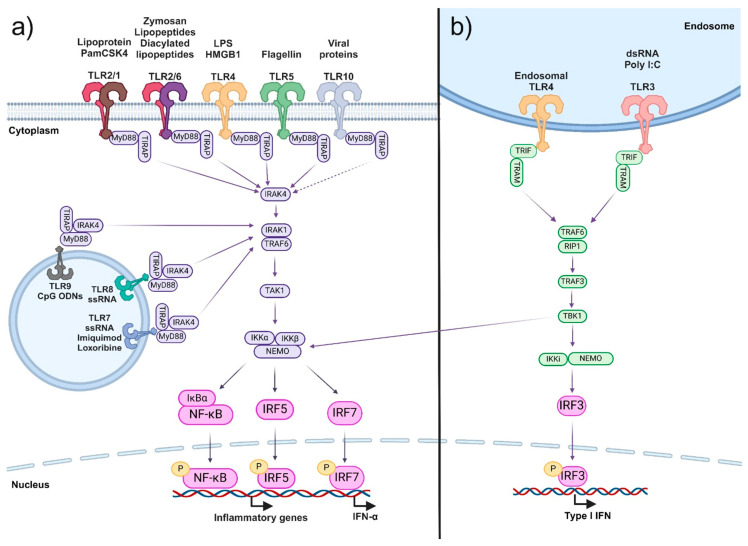
TLR-mediated signaling. Activation of TLRs can induce different signaling pathways. The most common signaling pathway induced by TLRs is the MyD88-dependent pathway (**a**). Although TLR10 has been shown to interact with MyD88, the complete signaling pathway has not been demonstrated experimentally. The MyD88-independent pathway is induced only by the activation of TLR4 and TLR3 (**b**). The signaling cascade culminates with the activation of transcription factors that, in turn, induce the expression of genes involved in various processes, including the immune response. Created with BioRender.com.

Following the recognition of their agonists, TLRs trigger a signaling cascade (Figure 1). The canonical signaling pathway induced by TLRs (other than TLR3) begins with the recruitment of an adapter protein called MyD88. Subsequently, the IRAK4 kinase is recruited to the MyD88–receptor complex, resulting in the phosphorylation and activation of IRAK1, which allows the recruitment of TRAF6. TRAF6 then activates the TAK1/Tab1/2/3 complex, which, in turn, phosphorylates and activates the IKK complex; ultimately, NF-κB is released from its inhibitor IκB. NF-κB is translocated to the nucleus, where it activates the expression of genes encoding molecules that participate in processes such as the immune response, survival, migration, proliferation, and apoptosis, among others. TLR4 can also lead to the activation of TRIF, culminating in the activation of transcription factor IRF-3, which, in turn, promotes transcription mainly of type I interferons. For its part, TLR3 also signals through TRAF3 but without the intervention of MyD88 [3,4,5].

Under homeostatic and pathological conditions, TLRs play important roles in nervous system processes, such as neurodevelopment, neurogenesis, cellular migration, differentiation, the neuroinflammatory response, and neurodegeneration [3,5]. TLRs are found in virtually all resident cells of the nervous system. TLRs are expressed on glial cells (microglia, astrocytes, and oligodendrocytes), neuronal progenitors, mature neurons, and cancer cells. Below, we present an overview of the expression of TLRs in the main cell types found in the nervous system and a brief description of the current evidence of their contribution to the function and maintenance of central nervous system (CNS) homeostasis.

## 2. TLR Expression and Function in Glial Cells and Neurons

A significant proportion of the brain mass is composed of nonneuronal cells called glial cells, which include microglia, astrocytes, and oligodendrocytes. In addition to providing neuronal support, some of their functions include regulating the proliferation, migration, and neurogenesis of neuroblasts; promoting axonal remodeling; secreting the extracellular matrix; modulating synaptic transmission by controlling neurotransmitter availability; and maintaining osmotic, ionic, and pH homeostasis. Additionally, these nonneuronal cells contribute to the provision of energetic and metabolic support to the cellular microenvironment, among other functions [6].

### 2.1. Microglia

Microglia are resident macrophages of the CNS that specialize in the host defense response through the detection of MAMPs and DAMPs by PRRs, the secretion of proinflammatory cytokines (IL-1β, TNF-α, IL-6, and IL-18) and chemokines, the phagocytosis of cellular debris and dead cells, and the maintenance of myelin [7]. Some evidence indicates that human microglia express TLR1–TLR9, and their activation induces different responses in these cells [8].

Microglia can acquire different functional phenotypes depending on the brain microenvironment. Two phenotypes are traditionally recognized: M1 (proinflammatory) and M2 (neuroprotective). When activated by PAMPs (such as LPS) or inflammatory factors (such as IFN-γ), microglia induce the M1 phenotype, characterized by the production of IL-1β, TNF-α, IL-6, IL-12, metalloproteases (MMPs), and inducible nitric oxide synthase (iNOS), among others, which contribute to the inflammatory environment and neurotoxicity. The M2 phenotype is induced by IL-4, IL-10, IL-13, and TGF-β and promotes the expression of factors involved in tissue repair and cellular homeostasis, such as FIZZ1, arginase 1 (Arg-1), CD206, and insulin-like growth factor 1 (IGF-1) [9,10]. Moreover, a study revealed a novel state in which microglia, which are activated simultaneously by proinflammatory (LPS) and anti-inflammatory (IL-4/IL-13) triggers, can generate a regulatory phenotype through immune pathways [11]. The functional phenotype or polarization of microglia is reversible, unlike that of other terminally differentiated body cells [12]. Recently, a study of M2-polarized microglia revealed that TLR9 activation using a conjugate of ODN 1826 with hyaluronic acid could alter the expression of immunomodulatory cytokines, reversing the antitumor M1 phenotype and improving the survival of an orthotopic glioblastoma model [13].

On the other hand, the activation of TLR4 by LPS has been found to suppress autophagic activity in primary cultures of murine microglia by activating the PI3K-AKT1 pathway and subsequently inhibiting FOXO3, contrary to the autophagic activity triggered by TLR4 activation in bone marrow macrophages [14]. The activation of TLR5 by flagellin induces the release of proinflammatory molecules, chemotaxis, and increased phagocytic activity, while coculture with neurons induces neuronal loss and apoptosis [15].

The activation of TLR7 by the let7 miRNA family revealed that members with a specific nucleotide sequence (UUGU) induced greater secretion of proinflammatory cytokines and the expression of molecules involved in antigen presentation (MHC-I and CD45) and chemotaxis in primary cultures of mouse microglia [16]. This finding is very interesting because it is one of the few reports of the activation of TLRs with endogenous agonists in the context of the CNS.

TLR10 has also been shown to participate in the polarization of microglia to the immunoregulatory M2 phenotype. A study demonstrated that treatment with the active metabolite of vitamin D3 in a human microglia cell line considerably increased the expression of TLR10 and typical cytokines of M2, such as IL-10. This effect was sustained even when the cells were simultaneously stimulated with LPS, suggesting the anti-inflammatory role of TLR10 [17].

### 2.2. Astrocytes

Astrocytes are the most abundant glial cells in the CNS and support neural activity. Their functions are the recycling of neurotransmitters, ionic balance, maintenance, and formation of the blood–brain barrier (BBB), as well as communication with other cell types in the CNS [18]. Like microglia, astrocytes adopt the A1/A2 nomenclature for the functional phenotypes they present. The A1 phenotype is involved in neuroinflammatory processes, and the A2 phenotype is considered neuroprotective. Classically, microglia can induce the A1 phenotype through IL-1β [19].

Astrocytes contribute to the immune response against viral infections in the CNS by expressing TLRs and producing cytokines and chemokines. Human astrocytes in embryonic culture have been shown to express TLR1–TLR10 mRNAs in response to ligands of TLRs. TLR3 activation with poly(I:C) in astrocytes leads to the production of cytokines, such as IL-6 and TNF-α, as well as chemokines, such as CCL2, CCL5, CCL20, and CXCL10. These cytokines and chemokines recruit and activate cells of the immune system, such as T lymphocytes, B lymphocytes, monocytes, and dendritic cells [20].

Several studies have analyzed the regulation of astrocyte function mediated by TLRs. Most of these studies focused on the expression or release of inflammatory mediators, such as cytokines and chemokines. For example, in a rat model of intracerebral hemorrhage (ICH), researchers found that aquaporin-2 (AQP2), an astrocyte water channel, is a positive regulator of astrocyte activation that promotes IL-1β expression via TLR4/NF-κB [21]. Another group performed studies with mouse spinal cord astrocytes focusing on the inhibition of TLR9 through its antagonist ODN 2088 and reported that in vitro stimulation with ODN 2088 caused a decrease in the proliferation and migration of astrocytes and modulated the release of chemoattractants, such as CCL1 and CCL2, and chemokines, such as CCL9, whereas in a murine model, treatment with ODN 2088 caused a decrease in reactive astrocytes and induced a neuroprotective environment [22,23].

### 2.3. Oligodendrocytes

The cells responsible for myelinating axons of the CNS are oligodendrocytes; these make the myelin sheath extend from the plasma membrane and supply energy to the axon [24]. The myelinating equivalent of these cells in the peripheral nervous system is Schwann cells, although these cells have been shown to invade the CNS under pathological demyelinating conditions as a rescue mechanism [25].

Most research on TLRs in oligodendrocytes has focused on their activation and myelinating function [26]. The TLR2 and STAT3/SOCS3 signaling pathways are associated with low expression of myelin genes, such as MOG (a protein related to myelin deposits of oligodendrocytes) [27]. TLR2 overexpression is also associated with cuprizone-induced demyelinating damage in a murine model, where a decrease in mature oligodendrocytes in the corpus callosum was also found after cuprizone intoxication [28]. In addition, the activation of TLR3 with poly(I:C) in primary cultures of mature murine oligodendrocytes decreased the expression of myelin genes (PLP1 and MOG) and increased the expression of CCL2 and CXCL10 [29].

The proliferation of oligodendrocyte progenitor cells (OPCs) can also be altered by the activation of TLRs. Blocking the interaction of human endogenous retrovirus type W with TLR4 rescued the maturation of OPCs into mature myelinating oligodendrocytes and decreased the secretion of proinflammatory factors (iNOS and IL-6) [30]. A similar effect was observed on traumatic brain injury (TBI), in which the postinjury release of HMGB1 caused a decrease in OPC proliferation through TLR2/TLR4, revealing the participation of these receptors in the regulation of OPCs [31].

However, the evidence has shown that Schwann cells not only participate in myelination but also promote immunomodulatory responses through TLRs. For example, a study of sciatic nerve injury in a murine model revealed that such injury results in the recruitment of Schwann cells and the secretion of proinflammatory factors (TNF-α, IL-1β, and IL-6) via TLR4/MD2 [32]. Similarly, the administration of 1-methyl-4-phenyl-1,2,3,6-tetrahydropyridine (MPTP) in a murine model of Parkinson’s disease causes the formation of p-α-synuclein deposits and an increase in TLR2 expression in enteric Schwann cells, promoting the production of proinflammatory factors (IL-1β, TNF-α, and NLRP3) through the TLR2/MyD88/NF-κB pathway, and the recovery of intestinal activity was achieved using a TLR2 inhibitor [33,34]. Despite the progress to date, more studies are required that address the function and expression of all members of the TLR family in the context of oligodendrocytes.

### 2.4. Neurons

Neurons are the functional unit of the nervous system. Their participation is not limited to information processing, as they also intervene in other processes, such as angiogenesis, the inflammatory response, proliferation, and neurogenesis, through the secretion of different factors and the modulation of different signaling pathways [35]. Interestingly, neurons express all TLRs in mice and humans, although their function has not yet been fully explored [36,37,38,39,40,41,42]. TLRs can trigger very diverse effects depending on the type of neuron and the context in which they are found. For example, TLR4 can modulate the feeding behavior of mice, as demonstrated by a TLR4 conditional knockdown mouse study on dopaminergic neurons of the ventral tegmental area, in which these mice reduced food reward behavior and suppressed the expression of tyrosine hydroxylase and dopamine at the neuronal level [43]. A study in primary cultures of murine hippocampal neurons revealed that activation of the TLR3/TRIF pathway reduced excitability by inhibiting Na^+^ channels and synaptic activity by reducing AMPA receptor expression; these results were similar to those in cultures of neurons derived from human pluripotent stem cells [44]. TLR9 can modulate the survival and fate of neurons, as shown in an in vivo model of sepsis-associated encephalopathy, in which mice in the treated group exhibited increased neurodegeneration and TLR9 expression in cortical neurons. The TLR9/p38 MAPK/ERK axis was shown to be associated with the activation of PANoptosis, while treatment with the TLR9 inhibitor ODN 2088 improved the survival rate of the mice [45].

In a model of peripheral nerve damage, murine dorsal root ganglion neurons (mDRGs) increased TLR7 expression after injury, and silencing TLR7 decreased hypersensitivity and chronic pain in mice [46]. Interestingly, TLR10 expression has also been demonstrated in human neurons. TLR10 mRNA expression is higher in primary cultures of neurons derived from fetal brains compared with neuronal cell lines, but its function has been poorly studied [47]. However, more studies are necessary to clarify its function.

## 3. Role of TLRs in the Blood–Brain Barrier

**Figure 2:**
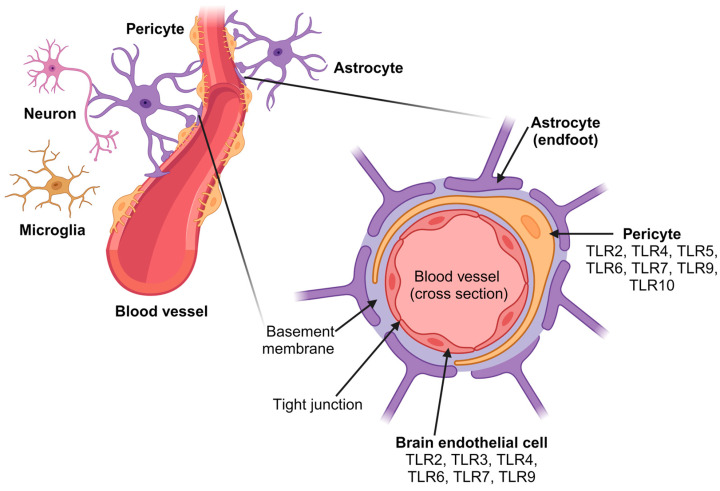
Schematic representation of the BBB located in the central part of the NVU. A cross-section of a blood vessel of the blood–brain barrier is depicted, showing the three main cell types that compose it (endothelial cells, pericytes, and astrocytes), other cell types of the neurovascular unit (microglia and neurons), and their respective TLRs that are expressed. The main function of the neurovascular unit is the formation of the blood–brain barrier and neurovascular coupling. Created with BioRender.com.

The blood–brain barrier (BBB) is an essential biological barrier for the functioning of the CNS. The BBB is a highly specialized and selective structure found in the brain that controls the exchange of substances between circulating blood and nervous tissue, limiting the access of potent harmful or toxic substances circulating in the blood, which allows the maintenance of homeostasis of the CNS microenvironment [48,49]. The BBB consists of a layer of endothelial cells (ECs) connected by tight junctions lining the blood vessels of the brain, pericytes, astrocytes, and other cellular components, including microglia, neurons, and the vascular basement membrane, which, in turn, constitute the structure of the neurovascular unit (NVU) [48,50]. The vascular basement membrane is a network formed by different extracellular matrix proteins, including laminin, an important protein related to the degree of vascular integrity of the BBB. Additionally, pericytes around blood vessels that are embedded within the vascular basement membrane and astrocytic end-feet that also cover the vascular basement membrane, connecting the rest of their cell bodies to neurons and microglia within the brain parenchyma, are responsible for the induction and maintenance of the integrity of the BBB in the NVU (Figure 2) [48,51].

### 3.1. Brain Endothelial Cells

Brain endothelial cells (BECs) differ from other vascular endothelial cells in the human body because they act as selective transport barriers composed of intimate cell junctions that determine their permeability [49,50,52]. BECs are involved in several physiological processes, including the regulation of blood flow, maintenance of vascular integrity, and immune responses in the CNS [53]. Therefore, BECs are at the forefront of CNS protection and respond to stress factors, such as inflammatory, ischemic, and infectious events, in which TLRs play an important role [49,52]. Oxidative stress and TNF-α positively regulate the expression of TLR2, TLR3, TLR4, and TLR6 in human brain endothelial cells (hCMEC/D3) and mouse brain endothelial cells. Moreover, TLR2 and TLR6 activation increases the permeability of the BBB by downregulating adherens junction proteins [52]. The expression of TLR3, TLR4, and TLR7 is increased upon activation with the ligands poly(I:C), LPS, and imiquimod, respectively, in human cerebral microvascular endothelial cells (hCMVECs). Furthermore, activation of TLR3 and TLR4 induces a proinflammatory response; in contrast, activation of TLR7 with imiquimod has an anti-inflammatory effect [54]. The expression of TLR2, TLR4, and TLR9 mRNAs was described in endothelial cells and brain tissue from a murine model after experimental cerebral ischemic injury [55]. Specifically, the most highly expressed gene was TLR2, and a global gene expression analysis was used to determine the regulation of TLRs after cerebral ischemia. Additionally, TLR2 knockout in mice has been shown to have a protective effect at the neuronal level by reducing the cerebral infarct volume. TLR2 signaling may contribute to ischemic brain injury by mediating the production of proinflammatory cytokines, such as TNF-α and IL-1β, through the NF-κB signaling pathway. Therefore, TLR2 inhibition contributes to neuroprotection, and TLR2 signaling promotes ischemic brain injury [56].

On the other hand, the activation of TLR3 and RIG-I (retinoic acid-inducible gene-I) with synthetic ligands induces the expression of IFN-β and IFN-λ in BECs. In turn, the release of interferons into the extracellular medium decreases HIV (human immunodeficiency virus) replication in infected macrophage cultures. HIV infection is widely known to compromise the integrity and permeability of the BBB, but BECs mount an innate immune response to HIV infection in the CNS through TLRs and the RIG-I receptor [57].

### 3.2. Pericytes

Pericytes are cells with elongations that extend around the vascular wall and are closely related to endothelial cells. Pericytes, also called mural cells, are specialized cells that provide structural support to help maintain the integrity and stability of blood vessels. They are involved in angiogenesis, vascular homeostasis, vessel maturation, and blood flow regulation [58,59]. Little is known about the expression of TLRs in pericytes; the mRNA expression of TLR2, TLR4, TLR5, TLR6, and TLR10 was detected under basal conditions, and TLR9 mRNA expression was detected after TNF-α treatment and IFN-γ and IL-1β induction in human brain vascular pericyte cells [60]. Furthermore, TLR2 expression was observed in the postischemic vasculature of aged rats [61].

TLRs have a multifaceted immunological function in pericytes due to their secretion of several types of chemokines and cytokines, as well as their ability to regulate immune cell trafficking and the expression of adhesion molecules [58]. For example, the activation of the TLR4 receptor upon stimulation with LPS and high mobility group box 1 protein (HMGB1) in human pericytes triggers proinflammatory and proangiogenic factors through the NF-κB signaling pathway [58]. Another study analyzed the inflammatory response of brain pericytes after infection with the Japanese encephalitis virus (JEV). This study detected the expression of TLR7 in a pericyte cell line infected with JEV. TLR7 signaling in pericytes after JEV infection, as well as that induced by the TLR7 agonist gardiquimod, contributed to the production of IL-6, RANTES, and PGE2, compromising the integrity of the endothelial barrier and leukocyte chemotaxis [62]. However, more research is needed to understand the roles of TLRs expressed in pericytes in the function of the BBB.

### 3.3. End-Feet of Perivascular Astrocytes

The terminal feet of astrocytes are essential for the formation and maintenance of the BBB; these layers cover the perivascular structure of the NVU and, in turn, most of the cerebrovascular surface by enveloping endothelial cells and pericytes with their terminal feet [63]. Astrocytes perform several physiological functions to maintain the integrity of the BBB, such as regulating cerebral blood flow by releasing vasoactive factors, such as nitric oxide (NO) and arachidonic acid, which act on smooth muscle cells in blood vessels; regulating permeability via tight junction proteins, such as claudin-5; removing waste and toxins from the brain through phagocytosis and active transport; maintaining ionic balance within the brain by removing excess potassium ions released during neuronal activity; and by taking up and recycling neurotransmitters released at synapses [63,64].

TLR2 mRNA expression is increased in hemorrhagic tissues in two different mouse models of intracerebral hemorrhage. The volume of brain lesions and neurological deficits were reduced in TLR2 knockout mice compared to the wild-type control in both models. Moreover, in TLR2 knockout mice, there was less damage to the integrity of the BBB, neutrophil infiltration, and proinflammatory gene expression in injured brain tissue compared to wild-type mice. These mechanisms indicate that TLR2 plays a principal role in ICH-induced brain injury [65]. Another finding in the context of ischemic stroke is the expression of MMP9 induced by HMGB1 via TLR4 in astrocytes and the mouse brain after cerebral ischemia [66]. TLR4 protein expression was significantly increased in an astrocyte oxygen–glucose deprivation model, which evaluated the neuroinflammatory effects of Z-guggulsterone after cerebral ischemic injury [67]. Moreover, TLR4 expression in astrocytes increased after middle cerebral artery occlusion (MCAO). TLR4-deficient mice exhibited a reduction in infarct size and improvements in neurological and behavioral outcomes after MCAO compared to those of control mice [68].

### 3.4. Other Cell Types of the NVU

Microglia and neurons also express TLRs, which play important roles in the function of the cerebral vasculature. Microglia play a dual role in promoting both protection and injury in brain injury caused by subarachnoid hemorrhage (SAH). Microglial activation after SAH is mediated by microglia-associated receptors, such as TLR4, the receptor for advanced glycation end products (RAGE), and myeloid cell-expressed receptor-1 (TREM-1). These receptors are activated by DAMPs released during SAH, which trigger the production of proinflammatory mediators and the release of cytokines, such as TNF-α, IL-1β, and IL-12. These inflammatory cytokines aggravate brain injury by triggering neuronal apoptosis, the disruption of the BBB, and brain edema [69]. It has been shown that inhibiting TLR4 activation could reduce the inflammatory response and mitigate the detrimental effects of activated microglia after SAH in mice [70]. In focal cerebral ischemia, TLR2 knockout mice exhibited reduced CNS damage compared to wild-type mice. These findings suggest that TLR2 in microglia contributes to stroke-induced CNS injury [71]. Together, these findings suggest that the regulatory pathways of responses induced by TLRs could be therapeutic targets for reducing the damage generated during cerebral vascular disease. However, more studies are needed to elucidate the cellular and molecular mechanisms mediated by TLRs expressed in the BBB.

## 4. Regulation of Neurogenesis by TLRs

Neurogenesis is the process by which new neurons are generated and integrated into the neural circuit. The evidence shows that neurogenesis occurs mostly during embryogenesis and decreases during early postnatal development and adulthood [72]. However, there is considerable evidence that neurogenesis occurs in the adult human brain in specific regions, like the dentate gyrus of the hippocampus and the lateral subventricular zone [72,73]. Neurogenesis in the adult phase has also been documented in various species, including rats, mice, songbirds, and nonhuman primates [72,74]. Neurogenesis has been postulated to play a role in memory and learning processes, as it is also a protective mechanism against stress-induced attrition of the brain [75].

In humans, neurogenesis starts midway through the first trimester in the ventricular zone, where these cells eventually migrate to their destination [72]. Neural progenitor cells (NPCs) divide to contribute to the generation of additional progenitor cells and differentiated neurons [76,77]. This intricate process occurs across various proliferative zones, culminating in the migration of neurons to their final destinations for long-term maintenance [77]. Concurrent sets of progenitors that later generate oligodendrocytes and astrocytes are maintained [76,77,78]. Notably, microglia are not derived from neural progenitors but rather from the hematopoietic lineage [78]. Neurodevelopmental processes include several key processes, such as neurogenesis, gliogenesis, cell death, synaptic pruning, and myelination, which collectively shape the intricate architecture of the developing brain [77]. TLRs participate in different brain and plasticity-related processes, including neurogenesis [79]. Different studies have shown that TLRs are involved in various steps of successful neurogenesis, such as NPC proliferation, migration, differentiation, maturation, and the integration of newly formed neuronal networks [79,80]. In mice, during the early stages of neurodevelopment, the activation of TLRs mainly affects neurogenesis, and in postnatal stages, it becomes involved in neuronal function, including memory, learning, and behavior. Furthermore, these effects have been shown to be related to the development of behavioral disorders and psychiatric disorders, such as seizures or schizophrenia-like behaviors [81,82,83].

Studies have shown that the activation of TLR2 contributes to the differentiation of NPCs into neurons via MyD88-dependent signaling and induces their differentiation into astrocytes but has no effect on cell proliferation or cell survival [84]. It has recently been discovered that neurogenesis in the adult brain is promoted by TLR2 in the neural stem cells (NSC) of the hippocampal dentate gyrus in rats [85]. Overexpression of TLR2 promotes the cell proliferation of NSC and increases cell numbers [85]. In the same way, neuronal activation via a heat-sensitive cation channel called TrpA1 also increases proliferation, and this effect depends on Toll-2 in Drosophila [86]. The deletion of TLR2 in NPCs results in heightened self-renewal and apoptosis, as well as a delay in neural differentiation and increased proliferation. Moreover, the absence of TLR4 in NPCs has been found to enhance neuronal differentiation without affecting NPC proliferation.

Notably, a significant decrease in TLR1 expression, which similarly impedes the maturation of oligodendrocyte precursor cells from NPCs, was induced by the absence of TLR2 and TLR4 [87]. Additionally, a potential correlation between TLR2 and the proliferation of neural stem cells (NSCs) after traumatic brain injury (TBI) has been suggested. In a rat model of TBI, TLR2 expression in the dentate gyrus increased post trauma, and on the third day, TLR2 expression peaked. Later, on the seventh day post trauma, its expression decreased, but it was increased compared with that in the sham group. In addition, increased proliferation of NSCs was observed post trauma [85]. In contrast, during mouse brain development, embryonic NPC proliferation is negatively regulated by TLR3 [88]. Heightened neuronal proliferation and differentiation were seen in the absence of TLR4, with the extent varying with sex and age. Specifically, young female TLR4 knockout mice showed increased neuronal production in both the ventral and dorsal hippocampus, whereas in young males, this was seen primarily in the ventral hippocampus [89]. Inhibiting TLR4 with neutralizing antibodies increases NPC self-renewal [84]. Furthermore, TLR4 has a crucial role in both neurodevelopment and the regulation of NPC proliferation and differentiation in disease settings [90,91]. TLR4 was shown to play an important role in neurogenesis by promoting neuroblast migration and increasing the number of new cortical neurons after stroke, despite its negative effect on proliferation in the subventricular zone [91]. Consistent with another experimental model of stroke, the evidence demonstrated that HMGB1-mediated TLR4 activation is necessary to maintain NSC proliferation and promote cell differentiation into neuroblasts, promoting their migration [92]. These findings indicate the importance of TLR4 as a therapeutic target in the modulation of neurogenesis after stroke. Moreover, stimulation of TLR3 and TLR4 curtails NPC’s capacity for self-renewal, yet deficiency promotes NPC proliferation [91,92,93].

Recent research unveiled the role of TLR5 in regulating the cell cycle by inhibiting proliferation in adult hippocampal NSCs and promoting neural differentiation [41]. Additionally, deficits of TLR5 in mice lead to impaired fear conditioning memory performance, indicating its vital role in the modulation of neurogenesis and suggesting it as a potential target for treating neurological disorders. TLR5 regulates contextual fear conditioning memory associated with adult hippocampal neurogenesis [41]. Neuronal activation of TLR3 with poly(I:C), TLR7 with CL075, and TLR8 with CL075/polydT negatively modulates neurite outgrowth, alters synapse formation in a cell-autonomous manner, and regulates neuronal differentiation [94]. TLRs can regulate slow cellular processes through the canonical MyD88 and ERK signaling pathways and gene expression, including cell renewal and NPC differentiation to neurons [84,88]. Moreover, enhanced NPC proliferation in the subgranular zone of the dentate gyrus was seen in the absence of MyD88, highlighting the essential role of TLR signaling components in NPC proliferation, even without exogenous TLR ligands. Similar to their impact on NPCs in the subependymal and subventricular zones, as well as in the hippocampus, TLRs also influence the proliferation of retinal progenitor cells [84,88,93,95]. In newborn MyD88 knockout mice, the cortical neuron density was increased, the neocortical thickness was reduced, and the numbers of microglia, astrocytes, oligodendrocytes, and proliferating cells were similar in these mice compared to wild-type mice [96]. In adult MyD88 knockout mice, the neuronal density in the neocortex and hippocampus was increased, while the neocortical thickness did not differ [96].

Cells with diminished activation or expression of TLR3, TLR7, and TLR8 display aberrant neuronal maturation [94,97]. Different experiments have revealed that TLR3 stimuli with poly(I:C) in cultured mouse and cortical and hippocampal neurons lead to reduced dendrite length and the modulation of dendrite morphology. These effects were TLR3-dependent since they were not observed when neuronal TLR3 was knocked down. In this experimental paradigm, TLR3 signaling was mediated by the MyD88 pathway rather than by TRIF [97,98]. Furthermore, through the manipulation of TLR7 expression levels, neuronal morphology is altered, and the addition of ssRNA and synthetic TLR7 ligands, such as CL075 and loxoribine, but not imiquimod, to cultured neurons specifically restricts dendrite growth via TLR7 [97]. The authors subsequently revealed, using MyD88-, IL-6-, and TNF-deficient neurons, the importance of IL-6 and MyD88 in the TLR7 track to restrict dendrite growth [97]. In comparison, the stimulation of TLR8 with a synthetic agonist inhibited neurite outgrowth and induced the death of cortical neurons in vitro. These effects of the agonist were not mediated through the canonical TLR-MyD88-NF-κB signaling pathway [99]. However, decreases in IRAK4 and IκBα, which are involved in TLR-MyD88-NF-κB downstream signaling, coincided with the inhibition of neurite outgrowth and neuronal apoptosis [99]. It is important to highlight that within mammalian brains, under homeostatic conditions (the absence of MAMPs and DAMPs), TLRs take part in the regulation of NPC proliferation, dendrite and axon growth, synapse formation, and neuronal morphology [77,94].

**Figure 3:**
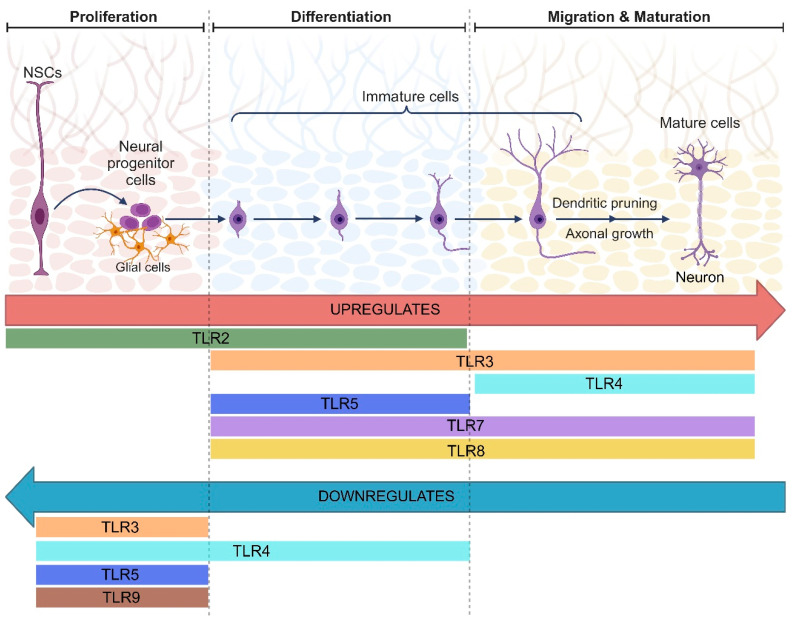
Roles and expression of TLRs in neurogenesis. Schematic representation of the principal stages of neurogenesis and the Toll-like receptors involved in the up- and downregulation of each process. Created with BioRender.com.

In summary, TLRs in the human brain regulate the proliferation and differentiation of neural progenitors and stem cells into neurons or glial cells. They also control cell survival, cell death, neurite growth and retraction, synaptogenesis, and the modulation of spine density and size (Figure 3).

## 5. Effects of TLR Misregulation on Behavior and Cognitive Function

TLRs have shown functional importance in behavior and cognitive function [96,100]. The absence of some TLRs results in behavioral abnormalities. For example, TLR2 knockout mice exhibit reduced anxiety, impaired sociability, aggression, and cognitive defects [100]. Similarly, the deficiency of MyD88 in mice results in specific behavioral traits, such as decreased locomotor activity and increased anxiety-like behavior. This highlights the diverse effects of TLR signaling pathways on behavior and cognitive function in animal models [96].

Another study has described TLR2 as a metabolic regulator of hypothalamic neurons that modulates feeding behavior and metabolic regulation [101]. Also, TLR3 knockout mice are less anxious than wild-type mice. TLR3-deficient mice, however, exhibit enhanced memory functions linked to the hippocampus [89]. Meanwhile, knockout TLR4 mice demonstrated improved spatial memory, which was particularly notable in young males and aged females, suggesting that it not only enhances memory but also modulates hippocampal inflammation, with effects varying by sex and age [102]. Interestingly, TLR4 deficiency was found to protect against sevoflurane-induced cognitive impairment and inflammation in mice [103]. Furthermore, TLR4 has been shown to play a significant role in cancer-associated cognitive impairment. It was found that the activation of TLR4 through cisplatin is a key mechanism leading to sphingosine-1-phosphate (S1P) production, resulting in cognitive dysfunction. S1P is a regulatory signaling molecule of vascular permeability, inflammation, angiogenesis, cancer growth, metastasis, and brain and cardiac development that contributes to the development of cancer-related cognitive impairment [104].

Recent investigations in organotypic slice cultures of rat hippocampus have revealed that the activation of microglia through the simultaneous or individual administration of TLR2, TLR3, and TLR4 agonists can induce diverse levels of dysfunction within neuronal networks [105]. Similarly, another study found that the activation of microglia, marked by increased proinflammatory cytokines, as a consequence of repeated social defeat stress was linked to social avoidance behavior, which was mitigated in mice lacking TLR2/TLR4. These findings highlight the significance of innate immunity in the medial prefrontal cortex during repeated environmental stress-induced behavioral alterations [106]. Correspondingly, the use of an antagonist (OxPAPC) to block TLR2 and TLR4 effectively prevented the escalation of inflammatory responses in the hippocampus, triggered by a 24 h challenge with LPS in vivo [107]. In addition, TLR signaling is involved in the inflammatory pathways that contribute to mediating depressogenic effects in the context of major depressive disorder [108].

TLR7-deficient mice exhibit reduced exploratory behaviors, decreased anxiety, reduced aggression, increased olfaction, and impaired contextual fear memory [82]. In contrast, prenatal TLR7 activation in mice with imiquimod induced a phenotype of reduced anxiety-like behavior and fragmented social behavior, including social partners, circadian cues, and gonadal hormone fluctuations, but normal locomotor activity. All of these effects were accompanied by decreases in microglial density but increases in ramifications [109].

On the other hand, TLR9 knockout mice exhibited hyper-responsive sensory and motor phenotypes, with no alterations in learning or memory [110]. Furthermore, seizure-induced cognitive decline was intensified by TLR9 deficiency [111]. The brains of adult MyD88 knockout mice showed specific behavioral traits, suggesting that MyD88 is a key factor in neurodevelopment. Morphologic and cellular changes in the mouse brain, as well as altered natural and specific behaviors, result from its absence [96].

TLRs play key roles in a range of mental health conditions, including depression, stress, and schizophrenia, and in addictions such as alcohol and drug consumption. For instance, in a rat model of stress-induced depression called chronic mild stress (CMS), it was demonstrated that by reducing the gastrointestinal microflora, the elevated expression of TLR4/MD2 mRNA and protein and the elevated levels of inflammatory mediators were reversed in the prefrontal cortex [112]. Subsequently, investigations by the same research group revealed that CMS resulted in increased intestinal permeability, enhanced migration of bacteria into tissues, and activation of TLR-associated transcription factors, along with reduced antioxidant activity [113]. These findings confirm the importance of the intestinal–neuroimmune axis interaction.

Recently, chronic social defeat stress (CSDS) in mice led to depressive-like behaviors accompanied by a significant increase in hippocampal TLR4 protein expression. Treatment with fluoxetine, an antidepressant, reversed depressive-like behaviors and increased the expression of TLR4 and TNF-α in the hippocampus. Inhibiting TLR4 with TAK-242 blocked CSDS-induced behavioral despair without affecting social avoidance or anxiety-like behaviors. TLR4 knockout also prevented CSDS-induced behavioral despair and increased the expression of TNF-α in the hippocampus. These findings suggest that TLR4 plays a role in behavioral despair, and its inhibition may be a potential treatment for individuals experiencing depressive-like behaviors [114].

Moreover, individuals with stable chronic schizophrenia showed evidence of over-activation of the monocytic TLR4 signaling pathway. TLR4 hyperactivity was related to cognitive impairment and reduced white matter integrity. Additionally, the study identified a sluggish response of the TLR4/NF-κB/IL-1β signaling pathway to LPS stimulation in patients with stable chronic schizophrenia compared to healthy controls [115].

TLR7 expression was heightened in postmortem samples of the brains of individuals (humans) with alcohol use disorder [116]. The stimulation of TLR7 with R848 in mice resulted in a reduction in both preference and ethanol consumption, effects that persisted even after removing R848 [116]. Conversely, the pre-administration of R848 before drinking led to an increase in ethanol intake and preference. Notably, specific alterations in innate immune transcript levels across different brain regions, including increased expression of MyD88 and TRIF-dependent signaling, were triggered after acute TLR7 activation. The study underscores the relationship between maladaptive behaviors, in this case, excessive ethanol consumption, and the innate immune system [116]. Previous research has also demonstrated a direct correlation between signaling TLR pathways and alcohol intake. For example, LPS TLR4-activation was directly linked to increased alcohol consumption, whereas inhibition of the downstream signaling component IKKβ (MyD88-dependent) using siRNA resulted in reduced alcohol intake [117,118]. Similarly, the activation of TLR3 increases alcohol intake in mice. Repeated exposure to poly(I:C) and ethanol leads to a persistent increase in alcohol intake, which is dependent on TRIF signaling. This study identified TLR3 as a potential therapeutic target to reduce excessive alcohol consumption and treat substance abuse disorders [119]. In addition, TLR4 signaling has also been demonstrated to induce neuroinflammation and have a direct impact on the onset of addictions to substances such as alcohol and opioids. Blocking TLR4 or its downstream processes has been shown to diminish the reinforcing effects of opioids and alcohol, with implications for withdrawal and relapse-like behaviors [120,121]. These findings highlight the close and complex interaction between innate immunity and diseases of neuronal origin, emphasizing the potential of TLRs as targets for future studies on therapeutic approaches.

## 6. Involvement of TLRs in Neuroinflammation and Neurodegeneration

Inflammation is a physiological process by which the immune system responds to injury and infection. Neuroinflammation is characterized by the presence of reactive glia and infiltration of immune system cells, in addition to the release of several inflammatory mediators, including cytokines and free radicals. This response has the objective of protecting and repairing neuronal tissue, but persistent neuroinflammatory conditions and excessive oxidative stress lead to a decrease or loss of neuronal function (neurodegeneration), cell death, and the inhibition of regeneration [122,123].

During neuroinflammation, microglia and astrocytes that can produce cytokines (such as IL-1β, IL-6, TNF-α, and IFN-γ), chemokines, prostaglandins, and ROS are activated and proliferate. Subsequently, peripheral immune cells, such as monocytes, macrophages, and T and B cells, infiltrate the circulation [124,125].

Below, we analyze the role of TLR-mediated neuroinflammation in nervous system diseases.

### 6.1. TLRs in Bacterial and Viral Infections of the Nervous System

Persistent viral infection in the CNS can produce inflammation in different regions of the nervous system, for example, meningitis (meninges) and encephalitis (brain), caused by the production of various inflammatory factors, including cytokines. The exacerbated release of cytokines constitutes what is called a “cytokine storm”, which can result in neurodegeneration and, consequently, behavioral and neurological diseases [126,127]. Recent studies have highlighted the roles of TLRs in CNS viral infection. For example, in BV2 microglial cells infected with JEV, the activation of TLR2 and TLR6 is key to the production of inflammatory factors, such as TNF-α, CCL3, IL-1β, and iNOS, via the PI3K-AKT pathway [128]. Moreover, JEV induces let7a/b expression in N9 microglia, and let7a/b can activate the NOTCH-TLR7 pathway and induce NF-κB activation and cytokine production [129].

It has been shown that in a mouse model of experimental autoimmune encephalomyelitis (EAE), neuroinflammation increased via the TLR2/MyD88 pathway, in addition to increasing M1 microglial polarization and the production of proinflammatory cytokines, such as TNF-α, IL-6, IL-1β, and MCP-1 [130]. Subcutaneous administration of the TLR2/TLR4 antagonist naltrexone to mice with EAE has been shown to reduce the mRNA levels of IL-1β, NLRP3, IkBα, TNF-α, and IL-7 in the dorsal lumbar spinal cord [131]. Consistent with these findings, another study of EAE mice revealed that the attenuated expression of TLR4 and HMGB1 in astrocytes and microglia reduced inflammation and demyelination [132]. On the other hand, TLR3 deficiency in humans may predispose to the autoimmune and demyelinating manifestations that occur in acute disseminated encephalitis triggered by the herpes simplex virus (HSV) [133]. Furthermore, complications, such as encephalitis, that occur in some patients with Puumala hantavirus and HSV may be due to a heterozygous mutation in the TLR3 gene [134].

The envelope (E) protein of SARS-CoV-2 in a mouse model can trigger neuroinflammation and depression-like behaviors mediated by the activation of microglia and astrocytes through TLR2 in the CNS, while blocking TLR2 can alleviate E-induced neuroinflammation [135]. In murine models, exposure to the SARS-CoV-2 spike protein affects cognitive function and synaptic loss via microglial activation and neuroinflammation mediated by TLR4 activation [136]. Polymorphisms of the GG genotype of TLR4 2604G>A (rs10759931) are associated with a greater risk of developing cognitive impairment following SARS-CoV-2 infection than the GA genotype [136].

In a microglial model of the human immunodeficiency virus (HIV), TLR3 activation mediated by poly(I:C) effectively and dose-dependently inhibited HIV infection by triggering the IFN signaling pathway, inducing the expression of ISGs (IFN-stimulated genes; restriction factors involved in anti-HIV activities), and the production of chemokines (RANTES, MIP-1α and MIP-1β) [137].

On the other hand, polymorphisms of TLRs are involved in the susceptibility to infections; in this context, the TLR2 2477G/A polymorphism contributes to an increased risk of pneumococcal meningitis in the Caucasian population [138]. In Angolan children, the single nucleotide polymorphism TLR4 rs4986790 increases the susceptibility to bacterial meningitis caused by Haemophilus influenzae, the risk of ataxia, and neurological sequelae, while TLR9 rs187084 decreases the risk [139]. A meta-analysis revealed that the TLR9 polymorphism rs352140 is associated with a decreased risk of bacterial meningitis [140].

In a model of pneumococcal meningitis, the TLR2/TLR4 heterodimer regulates inflammatory and antibacterial mechanisms; in mice lacking both TLR2 and TLR4 inoculated intracerebroventricularly with Streptococcus pneumoniae, disease severity is increased, survival is significantly impaired, and the proinflammatory cytokines IL-1β, TNF-α, and IL-6 are decreased, while IFN-γ increases and might lead to long-term neurological problems [141]. Likewise, pretreatment with the intraperitoneal administration of TLR9 CpG ODN has been shown to have a protective effect on mice with Streptococcus pneumoniae infection, prolong survival, decrease the bacterial concentration in the systemic circulation and CNS, and promote major microglial proliferation [142]. In contrast, in patients and mice with pneumococcal meningitis, TLR7 was overexpressed. The TLR7 overexpression-induced model showed NF-κB activation and proinflammatory cytokine production, while TLR7 silencing suppressed inflammatory reactions and apoptosis and promoted cell viability, reducing brain damage caused by meningitis in mice [143].

### 6.2. Neurodegenerative Diseases and TLRs

Many studies have reported the participation of TLRs in the most common neurodegenerative diseases; a few examples are mentioned below. Alzheimer’s disease (AD) is a progressive neurodegenerative disease characterized by the accumulation of amyloid-beta (Aβ) peptides and phosphorylated tau in the medial temporal lobe and neocortical structures, in addition to neuroinflammation and oxidative stress [144]. In murine models of AD, TLR1 and TLR2 are overexpressed [145,146]. In addition, human brain tissue samples from patients with AD expressed TLR3 in microglia and endothelial cells associated with Aβ plaques [147]. Tau fibrils in AD mouse models induced the activation of TLR2/MyD88/NF-κB in microglia and stimulated neuroinflammation [148]. Systemic administration of the TLR2 agonist zymosan and lipoteichoic acid in an AD mouse model induced microglia-mediated neuronal death; therefore, microbial infections may facilitate neurodegeneration [149]. The activation of TLR3 by the agonist poly(I:C) can modulate early life stress-induced working memory impairments in adolescent mice. The intraperitoneal injection of poly(I:C) increased the mRNA levels of IL-6 and NF-κB but not oxidative stress and contributed to neuroinflammation, consequently decreasing working memory performance [150]. TLR2 inhibition in mice with tauopathy results in improved learning and spatial memory retention, and thus, TLR2 is a potential therapeutic target for AD [148]. Blocking TLR2 with an antibody increased Aβ phagocytosis by microglia and inhibited the inflammasome, suggesting that inflammatory microglia are less phagocytic [151]. Selective inhibition of the TLR2 pathway decreased neuronal apoptosis, inhibited the hippocampal activation of NF-κB and microglial inflammation, and protected cognitive function in an AD mouse model [152].

However, existing studies on the role of TLR2 in AD have yielded conflicting results; the deletion of TLR2 impaired learning ability and memory function, aggravated increased anxiety and depression, and enhanced the activation of astrocytes in AD mice [153]. Pretreatment with Pam3Cys and the monophosphorylated lipid A ligands TLR2 and TLR4 in a rat model of AD increased the release of TNF-α and CCL3, improved cognitive function, restored synaptic plasticity, and decreased Aβ deposits in the brains of patients treated with Aβ to the control level [154]. Pretreatment with an intraperitoneal injection of poly(I:C) in mice with AD reduced anxiety and depression, increased spontaneous activity, and improved memory deficits. In addition, poly(I:C) eliminated Aβ deposits, attenuated neuronal loss, and reduced the activation of microglia in the hippocampus of AD mice [155]. The administration of a TLR9 agonist before and after AD in mice eliminated short-term memory deficits and decreased Aβ deposits through the phagocytic activity of microglia [156].

Parkinson’s disease (PD) is the second most common neurodegenerative condition, and it is characterized by the presence of Lewy bodies and substantia nigra neuronal loss [157,158]. There are several studies that address the role of TLRs in PD; however, they are still inconclusive and, in some cases, contradictory. Increased expression of TLR4, which can promote neuroinflammation, has been observed in patients with PD [159]. Also, in patients with PD, neuroinflammation activated by microglia upregulated the expression of neuronal TLR2 and TLR4 to activate the p38/JNK pathway, leading to an increase in phosphorylation, the accumulation of α-synuclein proteins, and the shortening of neurites [160].

In a mouse model of PD, TLR2 was found to be overexpressed, and its depletion had a neuroprotective effect by reducing α-synuclein expression and neuroinflammation; therefore, the loss of dopaminergic neurons was reduced [161]. The absence of TLR4 reduces neuroinflammation and prevents striatal dopaminergic neuron loss and dopamine depletion associated with a PD mouse model [162]. In contrast, a recent study found that the absence of TLR4 resulted in a decrease in lysosomal autophagy (CD68 and p62) in microglia and facilitated the propagation of α-synuclein aggregates and the progression of the disease [163].

Similarly, in MPTP-treated mice, TLR7 and TLR8 were upregulated, and TLR8 deletion prevented the loss of locomotor agility and stability and ameliorated the emotional state of the mice. In addition, the double knockout of TLR7 and TLR8 reduced α-synuclein aggregate formation, T-cell activation and dendritic cell numbers, and, accordingly, reduced neuroinflammation and neurodegeneration [164].

The TLR3 rs3775290 polymorphism and TLR9 rs352140 polymorphism seemed to reduce PD susceptibility and may be protective factors in Chinese Han and Iranian populations, respectively [165,166]. In contrast, the TLR4 rs4986791 and rs1153889 polymorphisms were associated with an increased risk of PD, while the interaction between rs1927914 and alcohol consumption was associated with a decreased risk of PD in the Chinese Han population [167]. Further studies are needed to clarify the participation of TLRs in the pathogenesis of PD.

## 7. Conclusions and Perspectives

Beyond their functions in responding to infections or damage, TLRs play an important role in processes such as neurogenesis, cognition, and behavior, revealing the close connections that exist between the nervous system and the immune system (Table 1). However, despite the large number of published works, many of the mechanisms are unknown, or the results are controversial and, therefore, not decisive. More studies are needed to determine the expression and involvement of TLRs in nervous system functionality in the context of humans specifically. There are both differences and coincidences in the expression, activation mechanisms, and signaling of TLRs between humans and other mammals, which complicates the interpretation of the results obtained and means that the findings of animal models need to be interpreted with caution.

Finally, it is important to highlight the lack of knowledge about the participation of endogenous agonists of TLRs that are not associated with damage or infections during neurodevelopment, which is a great area of opportunity for future research. Endogenous ligands could be the key to both the diagnosis and treatment of different neurodevelopmental and neurodegenerative diseases.

## Figures and Tables

**Table 1:** Summary of TLR participation in the nervous system.

Receptor	Outcome	Related Disease	Model	Refs.
**TLR1**	Increased receptor expression.	Epilepsy	Mouse model	[36]
**TLR2**	Negatively regulated maturation of oligodendrocyte precursor cells.	Multiple sclerosis	KO mouse	[26]
	Receptor deficiency decreased neuronal death, inflammation, and brain injury.	Cerebral ischemia	KO mouse	[37,56,71]
	Increased receptor expression.	Amyotrophic lateral sclerosis	Postmortem human spinal cord sample	[38]
	Receptor activation increased transendothelial permeability.	Not	Rat and human cerebral endothelial cells	[52]
	Receptor deficiency reduced MMP-9 activation in astrocytes.	Intracerebral hemorrhage	KO mouse	[65]
	Receptor deficiency induced schizophrenia-like symptoms.	Schizophrenia	KO mouse	[100]
	Regulated metabolic processes.	Obesity	Mouse neuron cell line	[101]
	Administration of antagonist decreased proinflammatory cytokines.	Multiple sclerosis	EAE mouse model	[131]
	Regulated neuroinflammation, depression-like behaviors, and dysosmia.	COVID-19	Mouse	[135]
	Receptor activation with tau fibrils induced stimulated microglial inflammation.	Tauopathy	Mouse	[148]
	Induced death of neurons and inflammation.	AD	AD mouse model	[149]
	Increased accumulation of α-synuclein proteins and the shortening of neurites.	PD	Dopaminergic neuronal cell line	[160]
**TLR3**	Increased receptor expression.	Multiple sclerosis	Human postmortem brain tissue	[8]
	Receptor activation decreased myelin expression and increased cytokine and chemokine production.	Maternal/fetal infection	Mouse oligodendrocyte precursor cells	[29]
	Receptor activation induced IL-1β secretion.	Viral infection	Human neurons	[39]
	Increased receptor expression.	Viral encephalitis	Human postmortem brain tissue	[40]
	Induced interferon expression.	HIV infection	Microvascular endothelial cells	[57]
	Negatively regulated NSPC proliferation.	Not	KO mouse	[88]
	Receptor activation increased alcohol intake.	Alcohol use disorder	Mouse	[119]
	Receptor activation in early stage decreased neuron loss and improved neurobehavioral functions.	AD	AD mouse model	[155]
**TLR4**	Increased receptor expression.	Multiple sclerosis	Human postmortem brain tissue	[8]
	Receptor activation inhibited autophagy and phagocytic function.	Not	Mouse microglial cells	[14]
	Induced neuronal death.	Cerebral ischemia	KO mouse	[37]
	Receptor deficiency decreased infarct size, improvements in neurological and behavioral outcomes, and inflammation.	Cerebral ischemia	TLR4-deficient mutant mouse	[66,68]
	Inhibited spatial memory and induced inflammation.	Not	KO mouse	[102,103]
	Receptor activation through cisplatin resulted in cognitive dysfunction.	Cancer	KO mouse	[104]
	Receptor deficiency decreased ethanol-induced impairment of BBB.	Alcohol use disorder	KO mouse	[121]
	Receptor blockade prevented detrimental effects on synapse and memory.	COVID-19	Mouse	[136]
	Receptor deficiency decreased inflammation, dopaminergic neuron loss, and autophagy and induced α-synuclein aggregate formation.	PD	KO mouse	[162,163]
**TLR5**	Induced inflammatory factors and neuronal apoptosis.	Not	Murine microglial and neuronal cultures	[15]
	Increased neuronal differentiation.	Not	KO mouse	[41]
**TLR6**	Activation of TLR2/TLR6 heterodimer increased interferon production.	HSV-1 infection	Mouse neuronal cell line	[42]
**TLR7**	Receptor activation induced microglial inflammatory cytokine production and antitumor effect.	Glioblastoma	Mouse glioblastoma	[16]
	Induced chemotaxis and prostaglandin production.	JEV infection	Pericyte cell line	[62]
	Receptor deficiency reduced anxiety, aggression, and contextual memory.	Not	KO mouse	[82]
	Acute activation of receptor reduced ethanol intake. Chronic activation induced tolerance to the adverse effects and an increase in ethanol consumption.	Alcohol use disorder	Mouse	[116]
	eceptor overexpression promoted meningitis.	Meningitis	Mouse	[143]
**TLR8**	TLR7 and TLR8 deficiency reduced astrogliosis, microgliosis, α-synuclein aggregate formation, and T-cell activation.	PD	TLR7/TLR8 KO mouse	[164]
**TLR9**	Induced antitumor effect.	Glioblastoma	Mouse microglial cell line	[13]
	Receptor inhibition decreased neurodegeneration.	Sepsis-associated encephalopathy	Sepsis rat model	[45]
	Induced TNF-α production in microglia and inhibition of seizure-induced aberrant neurogenesis.	Epilepsy	KO mouse	[111]
	Administration of ODN decreased bacterial burden in the cerebellum and bacteremia.	Meningitis	Mouse	[142]
	Receptor activation induced cognitive improvements and amyloid angiopathy reduction.	AD	AD mouse model	[156]
**TLR10**	Metabolite of vitamin D increased the expression of receptor.	Not	Human microglial cell line	[17]

KO, knockout; MMP-9, matrix metalloproteinase-9; EAE, experimental autoimmune encephalomyelitis; AD, Alzheimer’s disease; PD, Parkinson’s disease; HIV, human immunodeficiency virus; NSPC, neural stem and progenitor cells; HSV-1, herpes simplex virus-1; JEV, Japanese encephalitis virus.
